# Warning regarding hematological toxicity of tamoxifen activated CreERT2 in young Rosa26CreERT2 mice

**DOI:** 10.1038/s41598-023-32633-1

**Published:** 2023-04-12

**Authors:** Martina Rossi, Aude Salomon, Nicolas Chaumontel, Jenny Molet, Sabine Bailly, Emmanuelle Tillet, Claire Bouvard

**Affiliations:** 1 Laboratory BioSanté U1292, Univ. Grenoble Alpes, INSERM, CEA, 38000 Grenoble, France; 2 Univ. Grenoble Alpes, CEA, LETI, Clinatec, 38000 Grenoble, France

**Keywords:** Developmental biology, Genetics, Physiology

## Abstract

The Cre-lox system is a versatile and powerful tool used in mouse genetics. It allows spatial and/or temporal control of the deletion of a target gene. The Rosa26-CreERT2 (R26CreERT2) mouse model allows ubiquitous expression of CreERT2. Once activated by tamoxifen, CreERT2 will enter into the nuclei and delete floxed DNA sequences. Here, we show that intraperitoneal injection of tamoxifen in young R26CreERT2 mice leads to morbidity and mortality within 10 days after the first injection, in the absence of a floxed allele. Activation of CreERT2 by tamoxifen led to severe hematological defects, with anemia and a strong disorganization of the bone marrow vascular bed. Cell proliferation was significantly reduced in the bone marrow and the spleen resulting in the depletion of several hematopoietic cells. However, not all cell types or organs were affected to the same extent. We realized that many research groups are not aware of the potential toxicity of Cre recombinases, resulting in misinterpretation of the observed phenotype and in a waste of time and resources. We discuss the necessity to include tamoxifen injected CreERT2 controls lacking a floxed allele in experimental designs and to improve communication about the limitations of Cre-lox mouse models among the scientific community.

## Introduction

Genetically modified mice are commonly used in research, as animal models of human diseases or in order to elucidate the activity and function of genes in vivo. However, there are several hidden pitfalls one can encounter when using some of these models. This can lead to misinterpretation of the results and wrongful use of the animals. Here we report that tamoxifen injection in young Rosa26-CreERT2 (R26CreERT2) pups (P9 to P11) in the absence of a floxed allele results in severe toxicity and mortality.

The Cre-lox system is based on the ability of the bacteriophage P1 Cre-recombinase to catalyze the cleavage of a DNA sequence flanked by loxP sites. The expression of the CRE recombinase can be either under the control of a promoter that is active in a specific cell type or tissue, allowing tissue-specific deletion of the target gene (referred to as spatial regulation), or under the control of a promoter allowing ubiquitous expression of the Cre, and therefore ubiquitous deletion of the target gene. More recently, temporal regulation of the deletion of the target gene has been achieved using a Cre recombinase fused to a modified ligand binding domain of the estrogen receptor. This resulting CreERT binds to tamoxifen or its metabolites such as 4- hydroxytamoxifen (4-OHT), but not to estradiol^1^. In the absence of tamoxifen, CreERT is restricted to the cytoplasm. Administration of synthetic steroids, such as tamoxifen or 4-OHT allows steroid-mediated CreERT translocation into the nucleus, where it will recognize and excise floxed DNA sequences^2^. The CreERT2 was generated after the CreERT in order to increase the efficiency of the tamoxifen or 4-OHT induction^3^. These models are useful to circumvent developmental lethality. Several transgenic mouse lines expressing CreERT2 under the control of a ubiquitous or tissue specific promoter have been generated. In our case, for example, we needed a tamoxifen-inducible deletion in the whole body to study a gene that is required during development and expressed in several tissues and cell types. This can be achieved with the R26CreERT2 model, where CreERT2 is inserted into the Rosa26 locus, which is widely used for constitutive ubiquitous expression in mice^4,5^.

The potential side effects associated with tamoxifen administration are usually known and taken into account and tamoxifen-treated controls are commonly used^6^. However, it appears that the scientific community is not well aware of potential off-target effects due to Cre recombinase activity by itself, and Cre-positive controls without floxed alleles are lacking from most publications, including from our group. Mammalian genomes contain recombinase recognition sites called cryptic or pseudo loxP sites, which can be cleaved by Cre recombinase^7,8^. Cre expression in mammalian cells can cause DNA damage and reduce proliferation^9^. Steroid-inducible Cre recombinases were initially thought to be safer than constitutive Cre^10^, but it has been reported that tamoxifen activation of CreERT2 in adult mice resulted in cleavage of cryptic loxP sites, chromosomal abnormalities and transient hematologic toxicity^11^. In adult mice this toxicity is tolerated and animals can quickly recover. We previously showed that complete blood counts and blood chemistry panel of adult tamoxifen injected R26CreERT2 mice were normal^12^. However, the effects on young pups have not been reported. In the present study, WT and R26CreERT2 pups received intraperitoneal (IP) injections of tamoxifen from P9 to P11. We show that induction of CreERT2 by tamoxifen in young R26CreERT2 mice leads to severe toxicity, in the absence of a floxed target gene. We observed morbidity and mortality within ten days after the first injection. The pups stopped gaining weight and presented hematological defects with severe anemia and disorganization of the bone marrow vascular bed. We realized that many groups are still not aware of this problem and waste time and resources investigating a phenotype that is not due to the loss of the target gene, but that is caused by the recombinase itself. Many published studies using Cre/loxP transgenic mouse models do not include Cre-positive controls lacking loxP sites, and thus may have attributed roles to a target gene that were in fact due to Cre toxicity in part or entirely. We present here the toxic side effects observed and discuss the necessity to include appropriate controls in experimental designs and to report and communicate about the limitations related to transgenic models.

## Results

### Tamoxifen-induced CreERT2 activation in R26CreERT2 pups affects weight gain and survival with severe defects in hematopoietic organs

**Figure 1:**
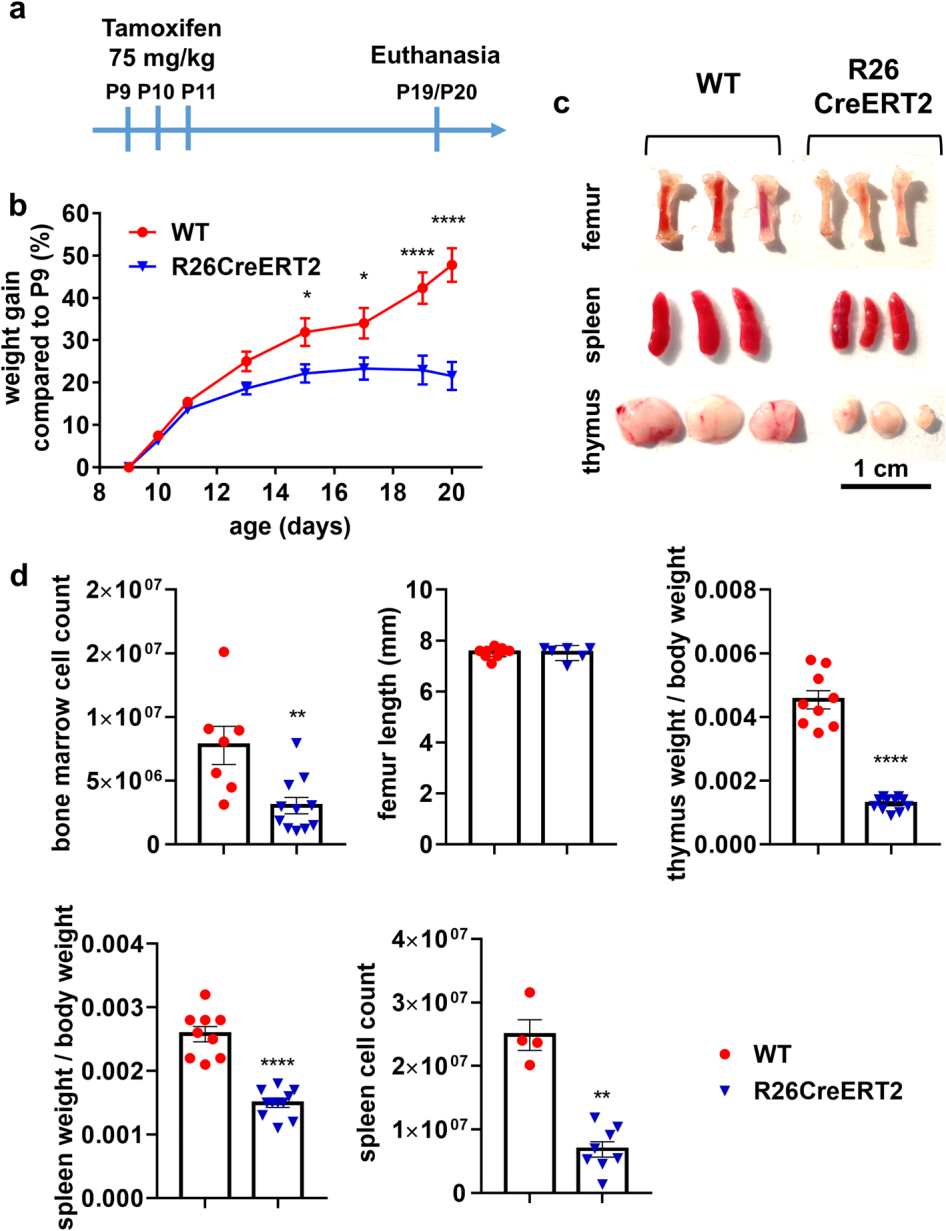
Tamoxifen-induced CreERT2 activation in pups affects gain weight and hematopoietic organs. (**a**) Experimental design for Cre ERT2 activation with tamoxifen injection. Litters are composed of WT and R26CreERT2 animals. Tamoxifen was injected IP at 75 mg/kg for 3 consecutive days from P9 to P11 and animals were euthanized at P19 or P20. (**b**) Weight curves of WT (n = 17) and R26CreERT2 (n = 27). Values are expressed as a percentage of weight gain compared to the weight at P9 (first tamoxifen injection). Two-way ANOVA with multiple comparisons was carried out to analyze differences in weight gain. (**c**) Representative images of femurs, spleens and thymus. 3 animals for each group are represented. Scale bar 1 cm. (**d**) Measurements of bone marrow cell count from two tibias and 2 femurs per animal, femur length, thymus weight, spleen weight, and number of cells per spleen (n = 4–11 mice per group). Mann–Whitney tests were used to assess statistical significance between two groups in these bar graphs. *p-value ≤ 0.05; **p-value ≤ 0.01; ***p-value ≤ 0.001; ****p-value ≤ 0.0001. Data are expressed as mean ± SEM of WT (red circle) and R26CreERT2 (blue inverted triangle) pups.

Without tamoxifen injection, R26CreERT2 pups and their WT littermates survive and grow normally and no obvious toxicity is observed (Fig. S1). However, when tamoxifen was injected IP (75 mg/kg) for 3 consecutive days from postnatal day 9 (P9) to P11, we observed that weight gain was reduced in WT animals compared to WT non-injected controls. More importantly, we observed that tamoxifen-injected R26CreERT2 animals stopped gaining weight after P15, which was not the case for their tamoxifen-injected WT littermates (Figs. S1 and 1a,b). This suggests that tamoxifen itself has a mild effect, which is usually taken into account, as tamoxifen-injected controls are commonly used. But it also shows that tamoxifen injection in Cre positive mice has a stronger effect, which is ignored by many groups, as tamoxifen-injected Cre positive controls without a floxed target are often missing in publications. This effect did not seem to be sex-specific as male and female mice were both affected (Fig. S2a). Therefore, male and female pups were pooled in all the other figures of the manuscript. Animal survival was affected in the tamoxifen-injected R26CreERT2 group, as 25 percent of the mice were dead at P19 (Fig. S2b). The remaining mice were not healthy, some of them presenting diarrhea, therefore the animals had to be euthanized at the latest at P20 in accordance with ethical guidelines. Autopsy revealed that the major hematopoietic organs were strongly affected in tamoxifen-injected R26CreERT2 pups, in comparison with tamoxifen-injected WT littermates: Bone marrow looked pale (Fig. 1c) with a reduced cell number although bone size was not affected (Fig. 1d). Spleen and thymus were significantly smaller (Fig. 1c,d). In addition, the color of the intestines was abnormal, with the presence of gas (Fig. S2c). It should be noted that tamoxifen-injected WT mice were normal (apart from a slight reduction in weight gain compared to non-injected controls), indicating that the observed phenotype was not induced by tamoxifen alone, but by the activation of CreERT2 by tamoxifen.

### Tamoxifen-induced CreERT2 activation in R26CreERT2 pups affects several blood cellular components and leads to severe anemia

**Figure 2:**
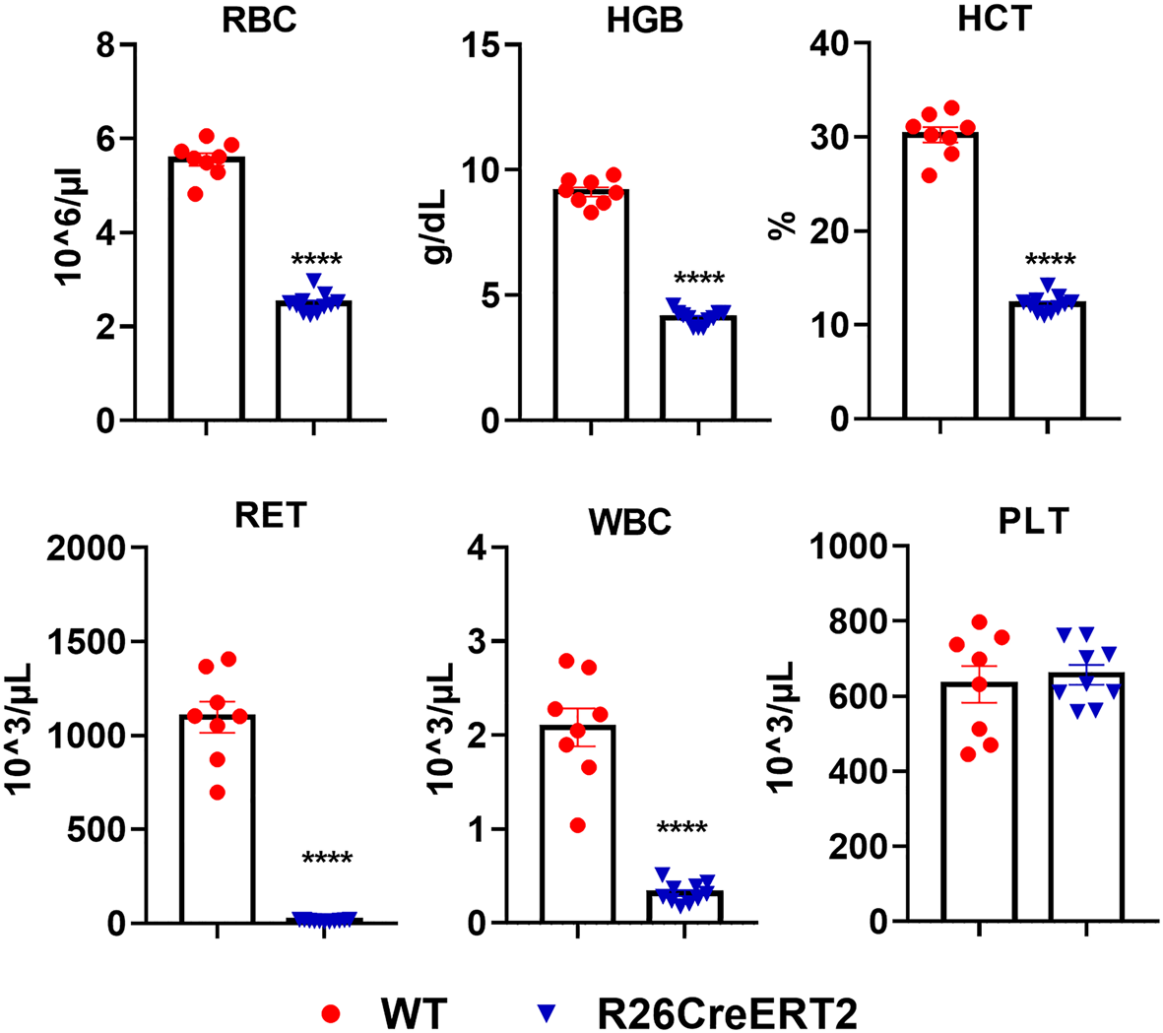
Tamoxifen-induced CreERT2 activation in pups affects several blood cellular components. Tamoxifen was injected IP at 75 mg/kg for 3 consecutive days from P9 to P11 and animals were euthanized at P19. Complete blood count analysis of WT (red circle) and R26CreERT2 (blue inverted triangle). *RBC* Red Blood Cells, *HGB* Hemoglobin, *HCT* Hematocrit, *RET* Reticulocytes, *WBC* White Blood Cells, *PLT* Platelets. Data are expressed as mean ± SEM of n = 8–11 mice per group. Mann–Whitney tests were used to assess statistical significance (**** p-value ≤ 0.0001).

Analysis of the whole blood at P19 (Fig. 2 and Table S1) revealed that activation of CreERT2 by tamoxifen leads to severe anemia, as red blood cell count (RBC), hemoglobin (HGB) and haematocrit (HCT) were strongly decreased. Reticulocytes (RBC precursors) were also totally depleted, indicating no compensatory blood cell production in order to reduce anemia. White blood cell count (WBC) was also significantly decreased. Within the WBC population, lymphocytes and neutrophils were depleted while monocytes were not. Platelet levels (PLT) of R26CreERT2 mice were not different from WT controls. Together, these results support that tamoxifen activation of CreERT2 strongly affects several specific blood cellular components.

### Tamoxifen-induced CreERT2 activation in pups affects several hematopoietic lineage-restricted precursors in bone marrow and spleen

**Figure 3:**
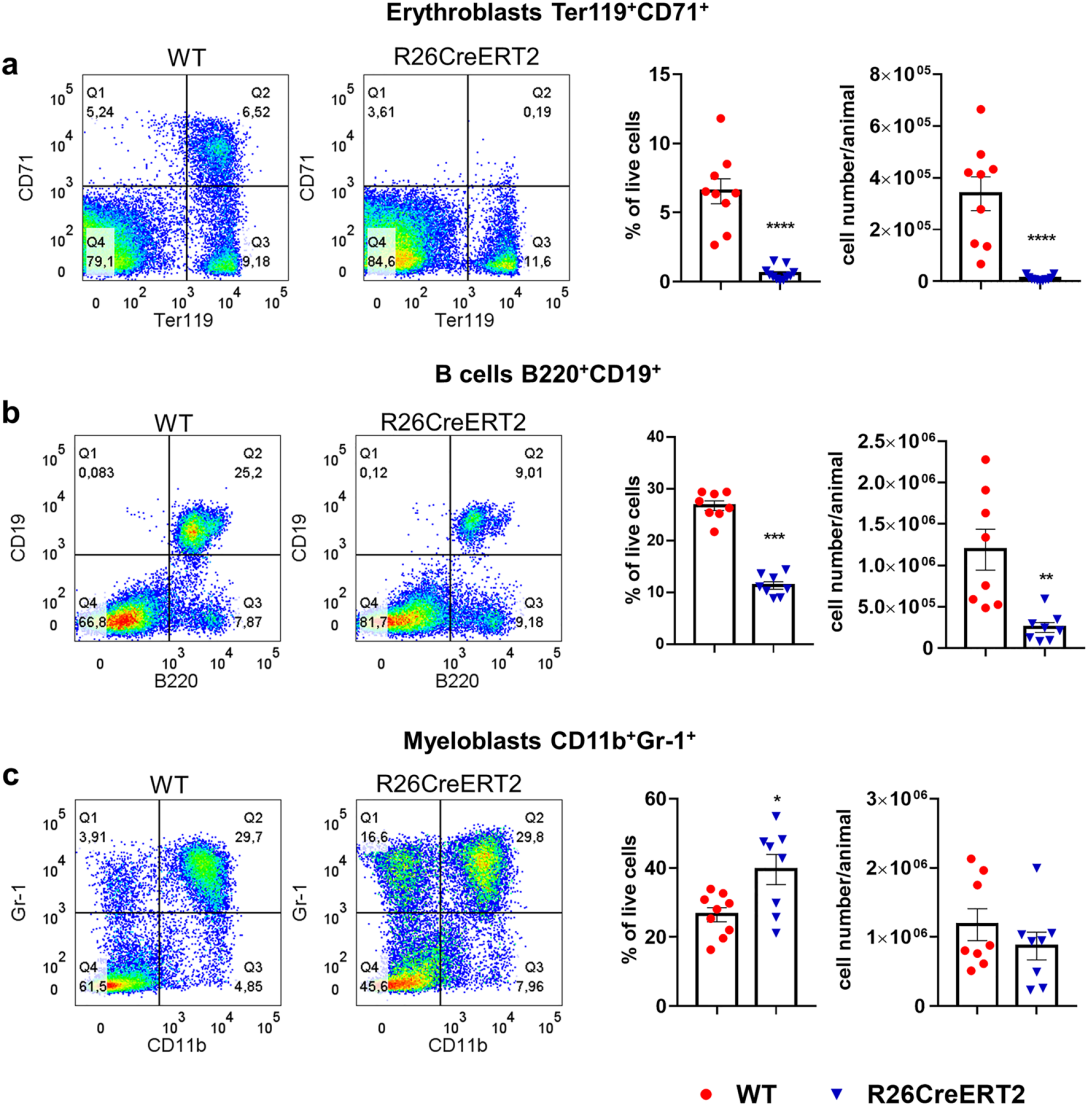
Tamoxifen-induced CreERT2 activation in pups affects lineage-restricted precursors in the bone marrow. Tamoxifen was injected IP at 75 mg/kg for 3 consecutive days from P9 to P11 and animals were euthanized at P19. Bone marrow from femurs and tibias from WT (red circle) and R26CreERT2 (blue inverted triangle) were analyzed by flow cytometry. Representative dot plots and quantitative analysis of Ter119 + CD71 + erythroblasts (**a**), B220 + CD19 + B cell precursors (**b**), and CD11b + Gr-1 + myeloblasts (**c**). Data are expressed as a percentage of the total live cells analyzed and as a number of cells per animal and are represented as mean ± SEM of n = 8–11 animals per group. Mann–Whitney tests were used to assess statistical significance (*p-value ≤ 0.05; **p-value ≤ 0.01; ***p-value ≤ 0.001; ****p-value ≤ 0.0001).

Cells found in the blood result from the differentiation of precursors that are present in the bone marrow and in the spleen. We used flow cytometry to quantify several precursors: Ter119^+^CD71^+^ erythroblasts (precursors for reticulocytes and then red blood cells), B220^+^CD19^+^ B cell precursors and CD11b^+^Gr-1^+^ myeloblasts (precursors for granulocytes and monocytes). In line with the results obtained from the blood samples, we observed that erythroblasts were totally depleted in the bone marrow and spleen of tamoxifen treated R26CreERT2 mice, compared to tamoxifen treated WT littermates, both as a percentage of cells and as a number of erythroblasts per mouse (Fig. 3 and Supplementary Fig. S3). B cell precursors were significantly reduced in both bone marrow and spleen, both as a percentage of cells and as a number of B cell precursors per animal (Figs. 3b and S3). The percentage of myeloblasts in the total cell population was decreased in the spleen but increased in the bone marrow, probably due to the loss of other cell types in the bone marrow (Figs. 3c and S3). The number of myeloblasts per animal was significantly reduced in the spleen, but the reduction was not statistically significant in the bone marrow (Figs. 3c and S3).

### Tamoxifen-induced CreERT2 activation in R26CReERT2 pups results in a strong disorganization of BM sinusoidal vessels

**Figure 4:**
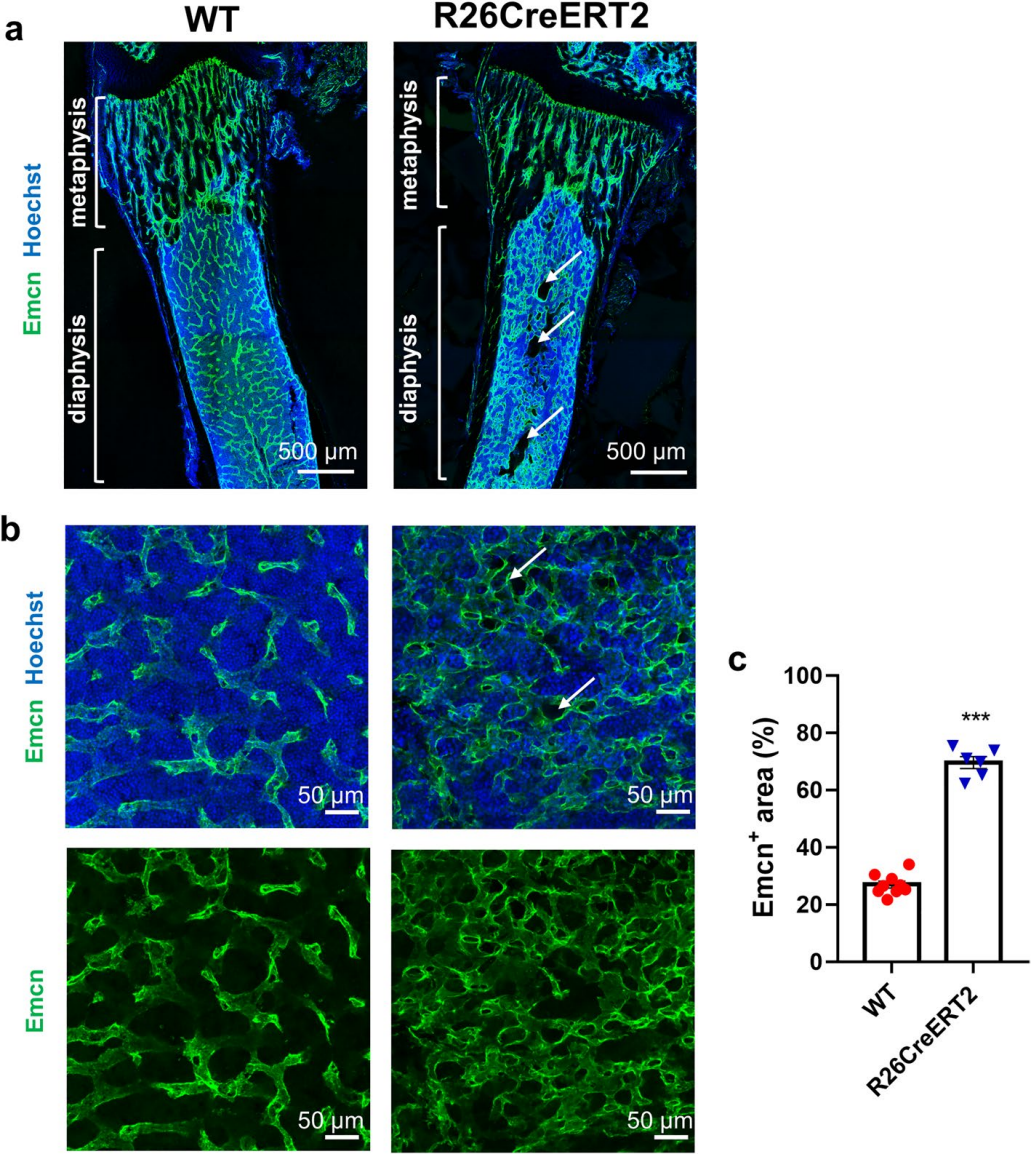
Tamoxifen-induced CreERT2 activation in pups results in a strong disorganization of sinusoidal vessels within the diaphysis of the bone marrow. Tamoxifen was injected IP at 75 mg/kg for 3 consecutive days from P9 to P11 and animals were euthanized at P19. Representative tile-scan images of femurs displaying both metaphysis and diaphysis (**a**, scale bars 500 µm), and representative single field confocal images of the bone marrow in the diaphysis (**b**, scale bars 50 µm) showing endomucin (Emcn, green) stained sinusoid vessels and cell nuclei stained in blue with Hoechst. White arrows are showing areas lacking hematopoietic cells. Quantitative analysis of the Emcn positive area, as a percentage of the total area of the field (**c**). Each data point represents the average of three different measurements per animal. Data are expressed as mean ± SEM of n = 6–9 mice per group. Mann–Whitney tests were used to assess statistical significance of differences between WT (red circle) and R26CreERT2 (blue inverted triangle) tamoxifen injected pups (***p-value ≤ 0.001).

Hematopoietic and endothelial cells of the bone marrow are tightly linked from early development through the adulthood. The vascular niche supports hematopoiesis in the bone marrow^13,14^. In order to assess the state of the bone marrow vasculature, P19 femur sections were stained with an antibody against endomucin (Emcn), a marker expressed by venous and capillary endothelial cells. The vascular network of the diaphysis of the bone marrow in tamoxifen-treated R26CreERT2 mice was dramatically disorganized, while no obvious defects were observed in the column-like vessels of the metaphysis (Fig. 4a). In the diaphysis, several areas of the bone marrow were devoid of hematopoietic cells, appearing as black holes in the images (Fig. 4a,b), while the vascular area in the bone marrow was strongly increased, with dilated sinusoids forming an abnormal network (Fig. 4b,c).

### Tamoxifen-induced CreERT2 activation decreases proliferation in the bone marrow and spleen of R26CreERT2 pups

**Figure 5:**
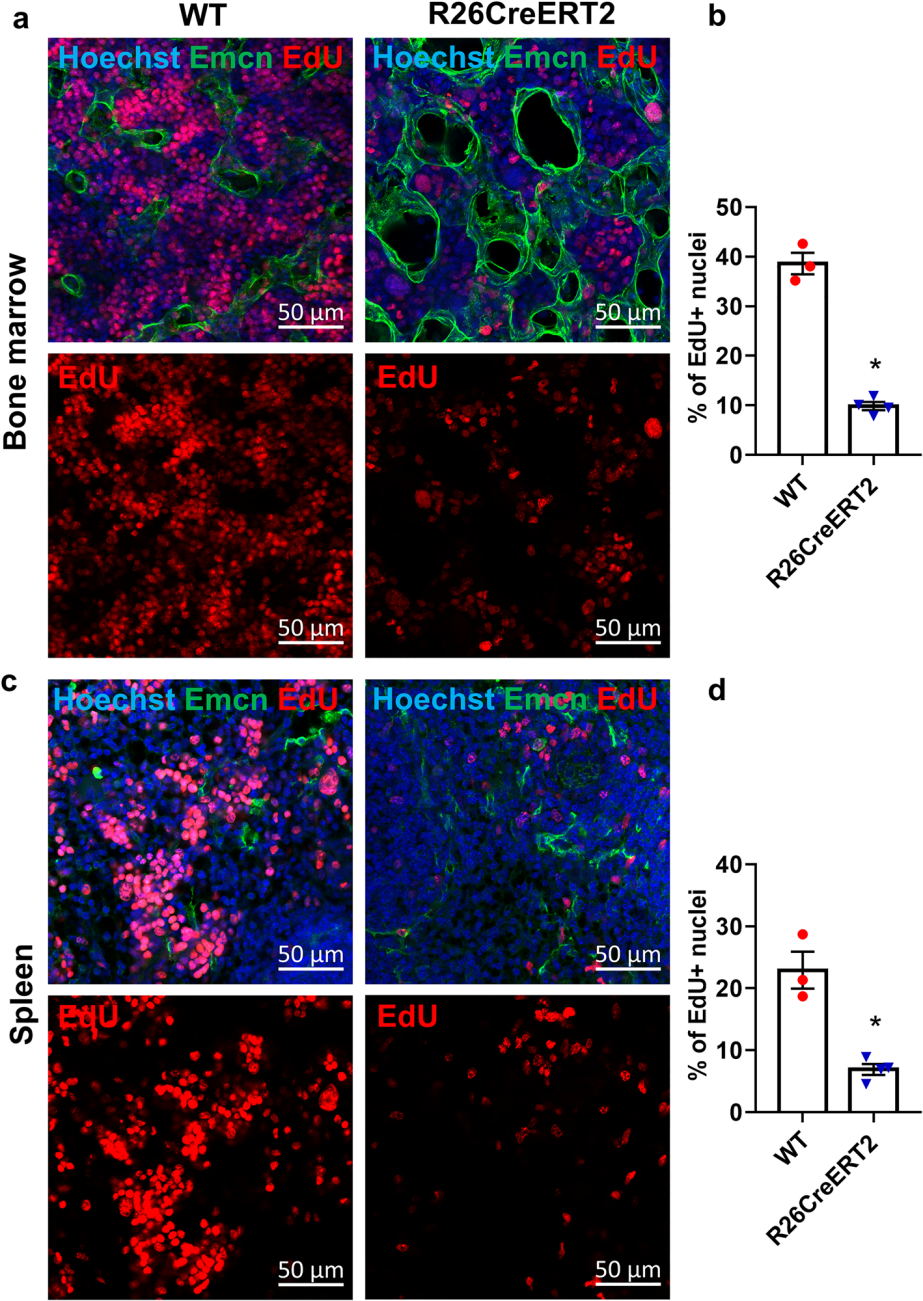
Tamoxifen-induced CreERT2 activation in pups results in loss of EdU + proliferative cells in bone marrow and spleen. Tamoxifen was injected IP at 75 mg/kg for 3 consecutive days from P9 to P11 and Edu was injected IP at P17 and animals were euthanized 3 h later. Representative images and quantitative analysis of the percentage of Edu + proliferative cells in bone marrow (**a**, **b**) and spleen (**c**, **d**) of WT (red circle) and R26CreERT2 (blue inverted triangle) pups. Endomucin (Emcn) staining (vessels) is represented in green, Edu + proliferative cells are represented in red, and nuclei in blue. Data are expressed as mean ± SEM of n = 3–4 per group. Mann–Whitney tests were used to assess statistical significance (*p-value ≤ 0.05). scale bars 50 µm.

It has been shown previously that Cre can reduce proliferation^9^. To assess proliferation in our pups, we performed an Edu incorporation assay at P17. We observed a strong reduction of proliferation in hematopoietic organs such as the spleen and the bone marrow of tamoxifen-treated R26CreERT2 mice, compared to tamoxifen-treated WT controls (Fig. 5). This decreased cell proliferation in hematopoietic organs could explain why several precursors and cell types are depleted in these organs and in the blood. We also analyzed other tissues, such as intestine liver or lung, but we found no obvious differences in cell proliferation between R26CreERT2 and WT mice in these organs (Fig. S4).

### Tamoxifen-induced CreERT2 activation increases the number of CD41 + megakaryocytes in the bone marrow of R26CreERT2 pups

**Figure 6:**
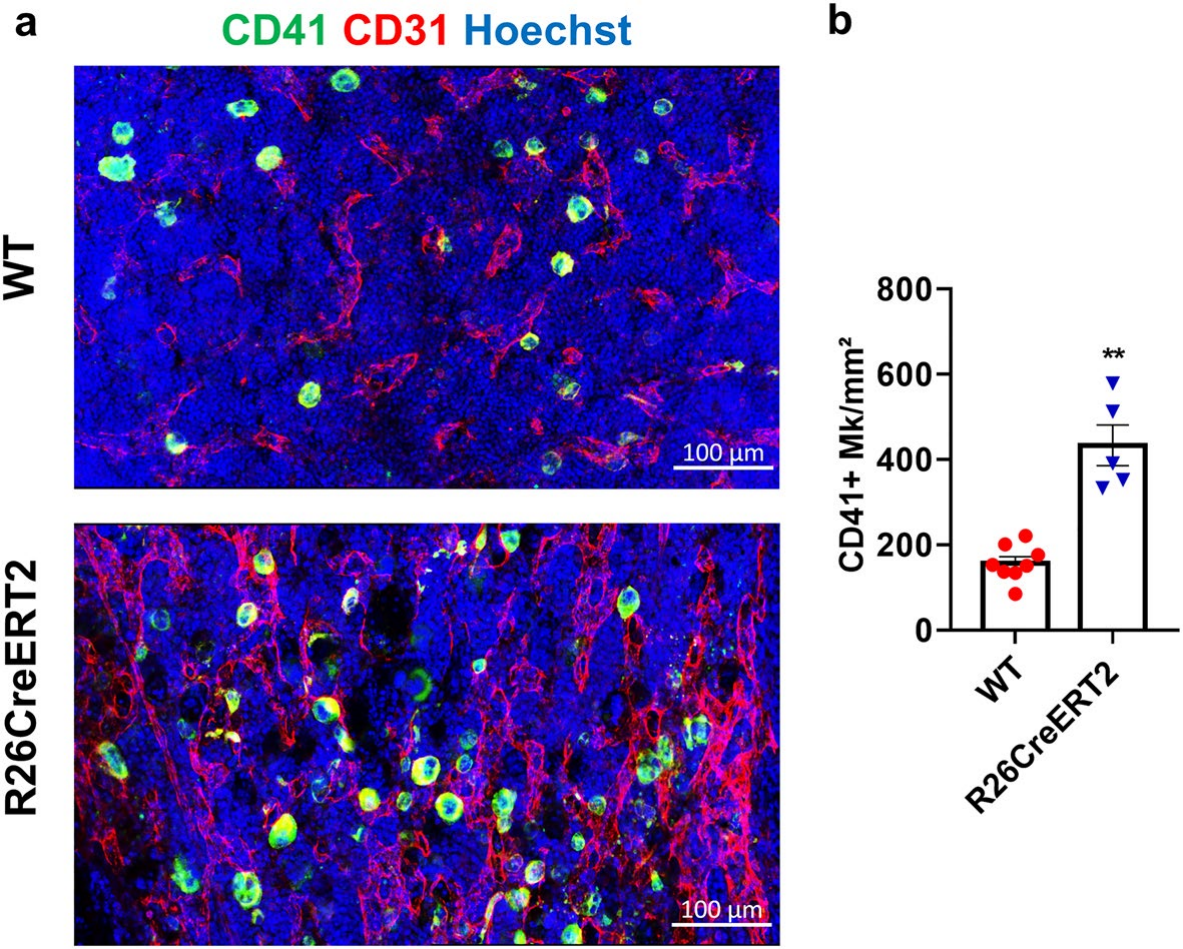
Tamoxifen-induced CreERT2 activation in pups results in an increase in CD41 + megakaryocytes in the bone marrow. Tamoxifen was injected IP at 75 mg/kg for 3 consecutive days from P9 to P11 and animals were euthanized at P19. (**a**) Representative images of bone marrow sections of WT (red circle) and R26CreERT2 (blue inverted triangle) pups. CD41 + megakaryocytes are stained in green, CD31 + vessels in red and nuclei in blue. (**b**) Quantitative analysis of the number of CD41 + megakaryocytes per square millimeter. Data are expressed as mean ± SEM of n = 5–8 mice per group. Mann–Whitney tests were used to assess statistical significance (**p-value ≤ 0.01). scale bars 100 µm.

Interestingly, CreERT2 activation did not affect platelet counts in blood, whereas WBC and RBC counts were strongly reduced. We thus wanted to study megakaryocytes (MK), which are responsible for producing platelets. Immunostaining of the bone marrow using an anti CD41 antibody to identify cells from the megakaryocytic lineage revealed an increase in the number of CD41 + cells in R26CreERT2 tamoxifen treated mice, compared to WT tamoxifen treated controls (Fig. 6).

### Decreasing the dose or the frequency of tamoxifen injection can decrease off-target hematological toxicity, but it also decreases on-target Cre-lox recombination efficiency

**Figure 7:**
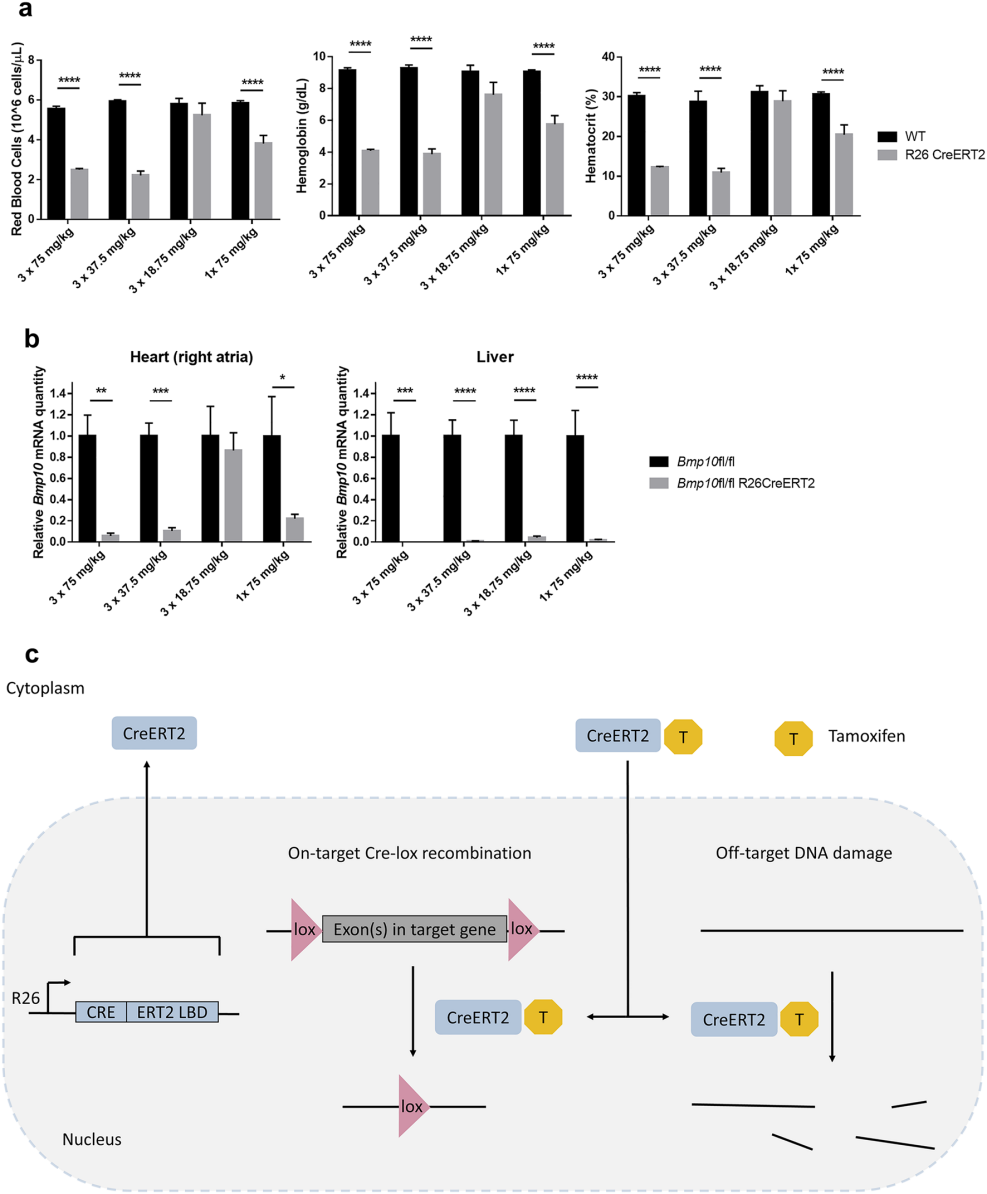
Decreasing tamoxifen dose or frequency of administration can reduce off-target hematological toxicity but it also decreases on-target Cre-lox recombination efficiency (**a**) WT and R26CreERT2 received intraperitoneal injection of tamoxifen at 75 mg/kg, 37.5 mg/kg or 18.75 mg/kg for 3 consecutive days from P9 to P11 or only one intraperitoneal injection of tamoxifen at 75 mg/kg at P9. Animals were euthanized at P19 and blood was analyzed. Data are represented as mean ± SEM of n = 4–12 animals per group. Two-way analysis of variance was used to analyze differences between WT and R26CreERT2. ****p-value ≤ 0.0001. (b) *Bmp10*fl/fl and R26CreERT2 *Bmp10*fl/fl mice received intraperitoneal injection of tamoxifen at 75 mg/kg, 37.5 mg/kg or 18.75 mg/kg for 3 consecutive days from P9 to P11 or only one intraperitoneal injection of tamoxifen at 75 mg/kg at P9. Animals were euthanized at P19, and *Bmp10* mRNA level was quantified in cardiac tissue (right atria) and liver tissue. Data are represented as mean ± SEM of n = 5–10 animals per group. Two-way analysis of variance was used to analyze differences between *Bmp10*fl/fl and R26CreERT2 *Bmp10*fl/fl. *p-value ≤ 0.05; **p-value ≤ 0.01; ***p-value ≤ 0.001; ****p-value ≤ 0.0001. (**c**) Cre recombinase is fused to a modified ligand-binding domain of the estrogen receptor (ERT2 LBD). Cre ERT2 LBD transgene is inserted in the Rosa26 (R26) locus, allowing ubiquitous expression. The resulting protein, CreERT2, is restricted to the cytoplasm in the absence of tamoxifen. In the presence of tamoxifen or its metabolites (T), CreERT2 is translocated into the nucleus. Once in the nucleus, CreERT2 excises the target DNA sequence flanked by loxP sites (on-target effect) but it also induces DNA damage (off-target effect), independently of the targeted Cre-lox recombination.

In order to investigate whether a lower dose of tamoxifen or a reduced frequency of injection could reduce toxicity while maintaining an efficient recombination, mice were injected with either 75 mg/kg, 37.5 mg/kg or 18.75 mg/kg of tamoxifen for three consecutive days (P9, P10, P11), or they were injected only once (P9) with 75 mg/kg. We analyzed blood parameters such as RBC count, hemoglobin concentration or hematocrit to assess hematological toxicity in WT and R26CreERT2 mice (Fig. 7a). The recombination efficiency was evaluated using mice with a floxed allele. We compared R26CreERT2 *Bmp10*fl/fl mice, for which an exon of *Bmp10* is flanked by loxP sites, with *Bmp10*fl/fl mice lacking the Cre recombinase. *Bmp10* is expressed in the cardiac right atria at a high level and in the liver at a low level^12^. We quantified *Bmp10* mRNA levels in cardiac right atria and liver tissue at P19 by RTqPCR (Fig. 7b). Lowering the dose or the frequency of administration of tamoxifen reduced hematological toxicity, but also the efficiency of the knockdown. The regimen with 3 doses at 37.5 mg/kg show a similar toxicity as the regimen with 3 doses at 75 mg/kg, while the efficiency of knockdown was slightly lower in the right atria (89% compared to 94%, respectively). The regimen with 1 dose at 75 mg/kg was still toxic (although it was less toxic than 3 doses at 75 mg/kg) and the efficiency of the knockdown was reduced (*Bmp10* mRNA level in the cardiac right atria was only reduced by 63% whereas it was reduced by 94% with the 3 doses at 75 mg/kg regimen). The only non-toxic regimen was 3 doses at 18.75 mg/kg. However, with this regimen *Bmp10* mRNA level was not reduced in the cardiac right atria. Interestingly, all regimen significantly reduced *Bmp10* mRNA level in the liver, highlighting that recombination efficiency can differ between organs. Overall, recombination efficiency and toxicity seemed correlated and we could not find a non-toxic regimen that allows an efficient recombination for this mouse line.

## Discussion

The Cre/lox recombinase system has several advantages, including the ability to control Cre expression spatiotemporally through the use of mouse models whose genome can be constitutively or inducibly altered (temporal control) either ubiquitously or in a specific subset of cells (spatial control). It is thus a highly versatile tool for studying gene function. Nonetheless, this system has some limitations. In particular, the potential risks of Cre recombinase toxicity, which appears to be widely ignored. Our results show that administration of tamoxifen to R26CreERT2 pups, in absence of a targeted floxed allele, is toxic. Indeed, within few days after tamoxifen injection, these pups stop gaining weight and present severe anemia and diarrhea, with a significant reduction in spleen and thymus weight and hypocellular bone marrow. Some mortality is observed and the remaining mice have to be euthanized for ethical reasons ten to twelve days after the first tamoxifen injection. This toxicity is not due to tamoxifen alone, as it is not observed in tamoxifen treated WT mice, and it is not due to the presence of CreERT2 alone, as it is not observed in non-injected R26CreERT2 mice. In fact, it is due to the induction of CreERT2 by tamoxifen. Our results in pups are in accordance with previous reports showing anemia in adult mice or embryos^11,15^. However, we show here that the phenotype is more severe in pups than in adults as it leads to premature mortality. Analysis of these animals revealed that several lineage-restricted precursors in bone marrow and spleen were significantly depleted and proliferation in these organs was strongly reduced. Interestingly, not all precursors were affected to the same extent. Erythroblasts seemed to be more affected than B cell precursors and myeloblasts. In blood, the numbers of red blood cells and white blood cells were strongly reduced, while platelet counts were unchanged compared to WT littermates. Cell proliferation was dramatically reduced in bone marrow and spleen, but not in other tissues such as intestines, lung or liver. Overall, it seems that although CreERT2 is ubiquitously expressed in the R26-CreERT2 model, some tissues and cell types are more sensitive than others. In particular, the bone marrow is extremely sensitive. During the first few weeks after birth, long bones expand rapidly, blood vessels start to form morphologically distinct capillary networks, and hematopoietic cells proliferate to cope with growth in blood and marrow volume. In the metaphysis, the capillaries form column-like structures that support bone growth and, in the diaphysis, a dense vascular network of sinusoids is formed, surrounded by proliferating hematopoietic cells. At 3–4 weeks, bone growth decelerates, the main structures are established and hematopoietic stem cells transition into a hibernating state^14,16–19^. In tamoxifen-injected R26CreERT2 pups, no obvious defect was observed in bone growth and columnar capillaries of the metaphysis seemed normal. However, in the bone marrow (BM), in addition to a reduction in overall proliferation and number of hematopoietic cells, the sinusoids were completely disorganized, with an increased vascular area and enlarged lumens. The observed vascular defect could be a consequence of the depletion of hematopoietic cells in the bone marrow, or could be directly caused by recombinase toxicity. This disorganization of the vascular bed can in turn have effects on the surrounding cells in the bone marrow. Interestingly, the number of megakaryocytes (MK), which produce platelets, was increased. It has been shown that the majority of the MK reside directly at the sinusoid BM, and it was proposed that the vasculature could dictate the distribution of MK in the BM^20^. The hypervascularization observed upon CreERT2 activation could therefore support MK stability, explaining why we find more MK in the BM of tamoxifen injected R26-CreERT2 mice. It is also possible that levels of activated CreERT2 are lower in MK or that they are more resistant to DNA damage caused by recombinase than other hematopoietic cell types. The effect on the blood vessels of the BM and on MK has not been reported in previous studies on R26CreERT2 mice.

Other models with ubiquitous expression of CreERT or CreERT2 are available. Tamoxifen administration to Ubc-CreERT and CAG-CreERT2 mice led to anemia and reduction of cellularity of bone marrow and spleen. This was attributed to the deletion of the floxed target gene, but it could in fact be due to recombinase toxicity^21,22^. The results of these studies should be reanalyzed with tamoxifen treated Cre positive controls lacking floxed genes. Cre toxicity has also been reported in tissue specific models. Cre expression in postmeiotic spermatids, under the control of protamine 1 *Ptm1* promoter, results in sterility due to illegitimate chromosome rearrangement^23^. DNA damage and tetraploidy was observed in the epidermis of keratin5 and keratin14 promoter—driven Cre expressing mice^24^. Cre recombinase cardiotoxicity has also been reported in constitutive or inducible models using the α-myosin heavy chain (αMHC) promoter^25,26^. Glucose intolerance has been reported in pancreatic beta cell specific RIP-Cre mice, which raised concerns in the field of diabetes and suggested to revisit the conclusions of many studies that did not include Cre controls^10^. Toxicity has also been described in T cell specific models^27,28^. However, the discovery of Cre toxicity in one field does not necessarily come to the attention of researchers from other fields. In the field of angiogenesis, a recent letter from 2020 showed that tamoxifen-activated CreERT impairs retinal angiogenesis in neonates, independently of gene deletion^29^. This publication should entice researchers to start including Cre positive controls, which is not the current practice at least in the field of vascular development.

One way of avoiding misinterpretation is to be aware of the limitations of the model used. The risk of Cre toxicity having an impact on the study can depend on the recombinase and promoter used, genetic background, developmental stage, cell type or organ of interest, and on the protocol for tamoxifen administration (route, dose, frequency). Indeed, young mice seem more sensitive than adults. Differences in Cre toxicity among different cell types or organs can depend on the expression level of the recombinase, but also on tamoxifen biodistribution and concentration in the cell or organ of interest (for inducible models)^26,28,30^. It is necessary to report these off-target effects for each strain and each protocol and to communicate among researchers. These studies should be referenced in the mouse genome informatics database. Although some companies give general guidelines about the Cre/lox system, they should also directly warn customers that are purchasing a specific mouse strain in which Cre toxicity has been reported.

Different ways to reduce toxicity have been proposed, such as the use of self-deleting Cre expression vectors^31^ or optimization of the protocol to find the lower possible dose of tamoxifen that allows efficient target gene deletion. Administration of tamoxifen allows CreERT2 translocation into the nucleus. Once in the nucleus, CreERT2 can excise the target DNA sequence flanked by loxP sites (on-target Cre-lox recombination), but can also cleave DNA at cryptic sites (off target DNA damage) (Fig. 7c). Reducing the dose or frequency of administration of tamoxifen can reduce the amount of CreERT2 in the nucleus, therefore reducing off-target DNA damage. However, it can also decrease on-target Cre-lox recombination. With the example of the R26CreERT2 *Bmp10*fl/fl mouse, we could not find a non-toxic protocol that allows an efficient knockdown. This is just one example, the optimization should be performed with each mouse line, as recombination efficiency can depend on several factors (length of the floxed sequence, type of cell in which the target gene is expressed, expression level…), and as the level of knockdown required depends on the scientific question. More importantly, if the absence of Cre toxicity is not proven, appropriate Cre positive controls should be used, especially with young mice. With Cre/lox transgenic models, a very common breeding scheme is to cross mice homozygous for the loxP-flanked allele and heterozygous for the Cre transgene with mice homozygous for the loxP-flanked allele but lacking the Cre transgene. Half of the progeny will be Cre positive, the other half will be Cre negative and all will be homozygous for the loxP-flanked allele. It is especially convenient when working with pups, as controls and mutants come from the same parents and can be analyzed simultaneously. Adding a Cre positive control lacking a floxed allele can be complicated, and increases the overall number of mice used, however it has to be tested in order to avoid misinterpretation. Our work support that it is essential for young R26-CreERT2. Primarily because of the hematopoietic toxicity in the pups, and secondly because even if the cell type of interest does not show DNA damage, anemia and poor overall health could have an effect on the biological system of interest.

The use of animals in research is tightly regulated and the 3Rs rule (Replacement, Reduction, and Refinement) is one of the most important ethical principles. Although adding a Cre control group can increase the number of animals in a specific experiment, we believe that it can be necessary to answer the research question accurately. Moreover, reporting Cre toxicity issues and improving communication should reduce the overall number of animals used by preventing repetition and misleading interpretation.

## Methods

### Mice

Rosa26-CreERT2 mice (Gt(ROSA)26Sor^tm2(cre/ERT2)Brn^; MGI:3764519; provided by Pr P. Chambon, IGBMC, Illkirch, France) were maintained in a C57BL/6 background. The R26 locus was kept at heterozygosity for the CreERT2 mice in all experiments. The offspring genotypes were determined by PCR as previously reported^32^, Cre positive (R26CreERT2) and Cre negative (WT) mice were obtained in Mendelian proportions (1:1). Without tamoxifen injection, these mice were viable and fertile. To induce CreERT2 activation, in all figures but Fig. 7, pups received daily intraperitoneal (IP) injections of tamoxifen (T5648, Sigma) diluted in corn oil for 3 consecutive days from postnatal day 9 to postnatal day 11 at a dosage of 75 mg/kg, as recommended by the Jackson laboratory. R26creERT2 and WT control littermates were injected with tamoxifen and monitored simultaneously. In Fig. 7, we tested different doses (75 mg/kg; 37.5 mg/kg; 18.75 mg/kg) or frequency of tamoxifen administration (daily injections on 3 consecutive days from P9 to P11 or one injection only at P9). To compare recombination efficiency with different tamoxifen regimen, *Bmp10*fl/fl and R26CreERT2 *Bmp10*fl/fl mice were used (only in Fig. 7). They were generated as previously described^12^. Body weight was recorded every day during the injection period and then on alternate days until the day of euthanasia. Euthanasia was performed using an intraperitoneal injection of pentobarbital 180 mg/kg. Upon sacrifice, bones, spleen, and thymus were collected and weights of spleen and thymus were recorded. Mice were housed in a pathogen-free barrier facility, under 14-h light/10-h dark cycle and temperature-controlled environment with access to a standard diet and water ad libitum. All animal experiments were performed in accordance with the relevant guidelines and regulations of Directive 2010/63/EU of the European Parliament on the protection of animals used for scientific purposes and the protocols were approved by an institutional ethics committee (CEtEA, CEEA44) and the study is reported in accordance with ARRIVE guidelines.

### Complete blood count analysis

Mice received an IP injection of pentobarbital 180 mg/kg and blood was collected by cardiac puncture in an EDTA-coated tube and analyzed on a Procyte Dx hematological analyzer (Idexx) within 2 h after sampling.

### Flow cytometric analysis

Bone Marrow (BM) single cell suspensions were obtained as described by Amend et al.^33^. Briefly, bones (femur and tibiae) were isolated, the metaphysis were cut and pooled bones of each mice were centrifuged at 5000 rpm for 30 s in a 0.5 mL tube with a perforated bottom placed into 1.5 mL tube. Collected cell pellets were resuspended in 1 mL ice-cold HBSS. Spleens were mechanically dissociated using a sterile plunger in 1 mL of ice-cold HBSS. BM and spleen cell suspensions were filtered through a 70 µm strainer. Red blood cells were lysed by the addition of RBC lysis buffer (Biolegend) and incubated on ice for 10 min. Cells were washed and resuspended in HBSS supplemented with 5 mM EDTA, 1% Fetal calf serum at 20 million cells/ml. Fc receptors were blocked by incubation with TruStain FcX PLUS (Biolegend) and 50 µL of cells were stained at 4 °C with the following conjugated antibodies CD71-PE (clone RI7217), Ter119-FITC (clone TER-119); Gr-1-PE (clone RB6-8C5), CD11b-FITC (clone M1/70); CD19-PE (clone 6D5), B220-FITC (clone RA3-6B2). All antibodies were from Biolegend and used at the recommended concentrations. Cells were stained with DAPI for dead cell exclusion. Cells were analyzed on a FACSMelody flow cytometer and data were analyzed with the FlowJo software.

### Bone sample preparation

Mice were sacrificed either at postnatal day 17 for proliferation assay or at postnatal day 19 for bone vasculature structure assessment and prepared for immunofluorescence analysis as described by Kusumbe et al.^34^. Freshly dissected long bones were fixed in 10% formalin solution (HT5011, Sigma) for 4 h followed by decalcification in 0.5 M EDTA for 24 h under gentle agitation at 4 °C. Bones were then washed in PBS and incubated in 20% sucrose and 2% PVP in PBS for 24 h at 4 °C. Bones were embedded in 8% gelatin, 20% sucrose 2% PVP in PBS and stored at − 80 °C for at least 24 h prior to sectioning.

### Immunostaining and confocal imaging

Embedded bones were sectioned at 100 µm thickness using a cryostat from Leica (CM3050 S). Sections were hydrated using PBS and permeabilized using 0.5% Triton-X100 in PBS for 15 min at room temperature (RT). Samples were then incubated in blocking solution (0.3% Triton, 1% BSA, 2% donkey serum in PBS) for 30 min at RT prior to primary antibody staining. Primary antibodies were diluted in blocking solution, incubated O/N at 4 °C. Species-specific secondary antibodies (Jackson) diluted in blocking buffer were added and incubated for 2–3 h at RT. Nuclei were stained with Hoechst (1/1000 in PBS) and mounted using FluorSave mounting media. The following primary antibodies were used: rat monoclonal anti-Endomucin (sc-65495, Santa Cruz, diluted 1/200), goat polyclonal anti-CD31 (AF3628, R&D, diluted 1/100), rat monoclonal CD41 FITC‐conjugated (MWReg30, Biolegend, diluted 1/50). All secondary antibodies were from Jackson ImmunoResearch and diluted 1/200.

Images were acquired using a LSM 880 Airyscan Confocal microscope (Zeiss), and processed and analyzed using Zen (Zeiss) and Fiji softwares.

### Proliferation assay in vivo

EdU from Click-iT EdU Alexa-555 imaging Kit (C10638, Thermofisher Scientific) was prepared at 2.5 mg/mL in PBS. Seventeen-day old mice received an intraperitoneal injection of of EdU (0.3 mg per mouse). Mice were euthanized 3 h later and femurs, spleen, intestine, lung and liver were harvested for immunofluorescence analysis. Bone samples were processed as described above. Spleen samples were fixed in 10% formalin solution (HT5011, Sigma) for 4 h followed by overnight (O/N) at 4 °C in a 15% sucrose in PBS. The following day spleen samples were placed into a 30% sucrose in PBS O/N at 4 °C and subsequently mounted in OCT embedding compound and stored at − 80 °C. Intestines, lung and liver were fixed in 4% paraformaldehyde O/N at 4 °C and were dehydrated and embedded in paraffin. Sections were prepared at 10 µm thickness. Click-iT EdU Alexa-555 imaging kit was used as recommended by the manufacturer (C10638, Thermofisher Scientific). Nuclei were stained with Hoechst 33342 (1/1000). Anti-endomucin antibody (sc-65495, Santa Cruz, diluted 1/200) was used to stain vessels in bone marrow and spleen. WGA-FITC lectin (W834 Invitrogen, 5 µg/mL) was used to stain glycoconjugates in intestines, lung and liver. Images were acquired using a LSM 880 Airyscan Confocal microscope (Zeiss), and processed and analyzed with Zen (Zeiss) and Fiji softwares. The number of Edu positive nuclei and the number of total nuclei stained with Hoechst were analyzed using Fiji. For each mouse, three images per organ were analyzed and the average of the three different measurements was used for statistical analysis.

### Real-time quantitative polymerase chain reaction

mRNA was extracted from cardiac right atria and from liver tissue using a nucleospin RNA kit (Macherey, Nagel). Reverse transcription was performed using iScript kit from Biorad and quantitative polymerase chain reaction was performed using SsoAdvanced SYBR green kit from Biorad. The Delta-Delta Ct (∆∆Ct) method was used to obtain relative *Bmp10* expression levels (TCCATGCCGTCTGCTAACATCATC; ACATCATGCGATCTCTCTGCACCA), normalized to Rpl13a levels (CCCTCCACCCTATGACAAGA; TTCTCCTCCAGAGTGGCTGT).

### Statistical analysis

Statistical analysis was performed using GraphPad Prism software to assess differences between WT and R26CreERT2 mice. Two-way analysis of variance (ANOVA) was used for weight curve analysis and for multiple testing when different tamoxifen regimen were used. Log-rank Mantel cox test was used to analyze survival curves. Mann Whitney test was used for all other graphs. Differences were considered statistically significant for p < 0.05. All data are presented in mean ± SEM.

## Supplementary Information

Supplementary Information.

## Data Availability

Data is available upon written request to the corresponding author.
