# Supplementary material for: Warning regarding hematological toxicity of tamoxifen activated CreERT2 in young Rosa26CreERT2 mice
Source: Sci Rep. 2023 Apr 12;13:5976. doi: 10.1038/s41598-023-32633-1 (PMC10097815; doi:10.1038/s41598-023-32633-1)
Supplement: Supplementary file 1 — Supplementary Information. [file 41598_2023_32633_MOESM1_ESM.pdf]

DATA SUPPLEMENT

Supplementary figure legends

Figure S1. WT (●) and R26CreERT2 (▼) mice received intraperitoneal injections of tamoxifen at 75 mg/kg for 3 consecutive days from P9 to P11 (solid lines), intraperitoneal injections of vehicle (dashed lines) for 3 consecutive days from P9 to P11, or were not injected (dotted lines). Body weight was recorded from P9 to P20. Data are represented as mean ± SEM of n=5-21 animals per group. Two-way analysis of variance was used to analyze differences. * p-value ≤ 0.05; **** p-value ≤ 0.0001 vs WT non injected control group.

Figure S2. WT (●) and R26CreERT2 (▼) mice received intraperitoneal injections of tamoxifen at 75 mg/kg for 3 consecutive days from P9 to P11. **(a)** Body weight was recorded from P9 to P20 for males and females. Data are expressed as body weight or as a percentage of gain in body weight compared to the body weight at P9. Data are represented as mean ± SEM of n=7-15 mice per group. **(b)** Survival curves of n=18 WT and n=45 R26CreERT2. **(c)** Representative photograph of the intestines of WT and R26CreERT2 mice at P19. The arrow shows accumulation of gas in the intestines of R26CreERT2 mice. We used two- way analysis of variance to analyze differences in weight or weight gain and log-rank Mantel cox test to analyze survival curves. * p-value ≤ 0.05; ** p-value ≤ 0.01; *** p-value ≤ 0.001; **** p-value ≤ 0.0001.

Figure S3. WT (●) and R26CreERT2 (▼) mice received intraperitoneal injections of tamoxifen at 75 mg/kg for 3 consecutive days from P9 to P11 and were euthanized at P19. Cell suspensions obtained from the spleen were analyzed by flow cytometry. Representative dot plots (left) and quantitative analysis (right) of Ter119+ CD71+ erythroblasts, B220+ CD19+ B cell precursors, and CD11b+ Gr-1+ myeloblasts. Data are expressed as a percentage of the total live cells analyzed or as a number of cells per animal and are represented as mean ± SEM of n=6-11 animals per group. Mann-Whitney tests were used to assess statistical significance (** p-value ≤ 0.01; *** p-value ≤ 0.001; **** p-value ≤ 0.0001).

Figure S4. WT (●) and R26CreERT2 (▼) mice received intraperitoneal injections of tamoxifen at 75 mg/kg for 3 consecutive days from P9 to P11. At P17 they received an intraperitoneal injection of Edu and were euthanized 3 hours later. Representative confocal microscopy images of intestine, liver and lung sections. Nuclei were stained with Hoechst (blue), glycoconjugates were stained with WGA-FITC lectin (green), and proliferative cells with Edu Click-it A555 kit (red). Scale bar = 50 µm.

Supplementary table S1: Tamoxifen-induced CreERT2 activation in pups affects several blood cellular components. Complete blood count analysis of WT and R26CreERT2 pups. RBC = Red Blood Cells, HGB = Hemoglobin, HCT = Hematocrit, MCV = Mean Corpuscular Volume, MCH = Mean Corpuscular Hemoglobin, MCHC = Mean Corpuscular Hemoglobin Concentration, RET = Reticulocytes, PLT = Platelets, PCT = Plateletcrit, WBC = White Blood Cells, NEUT = Neutrophils, LYMPH = Lymphocytes, MONO = Monocytes, EO = Eosinophils. Values are mean ± standard deviation.

	WT (Mean ± SD)	R26CreERT2 (Mean ± SD)
RBC (10^6/µL)	5.53 ± 0.23	2.55 ± 0.2
HGB (g/dL)	9.17 ± 0.45	4.23 ± 0.2
HCT (%)	30.45 ± 1.75	12.39 ± 0.93
MCV (fL)	54.85 ± 1.38	48.96 ± 1.24
MCH (pg)	16.45 ± 0.33	16.39 ± 0.74
MCHC (g/dL)	30.75 ± 0.99	33.66 ± 1.14
RET (10^3/µL)	1161.85 ± 326.05	15.07 ± 4.3
RET (%)	23.78 ± 1.48	0.59 ± 0.19
PLT (10^3/µL)	631.5 ± 138.5	657.22 ± 79.73
PCT (%)	0.5 ± 0.07	0.59 ± 0.07
WBC (10^3/µL)	1.72 ± 0.61	0.32 ± 0.12
NEUT (10^3/µL)	0.33 ± 0.13	0.09 ± 0.03
LYMPH (10^3/µL)	1.27 ± 0.53	0.16 ± 0.06
MONO (10^3/µL)	0.04 ± 0.03	0.04 ± 0.08
EO (10^3/µL)	0.08 ± 0.03	0 ± 0

Figure S1

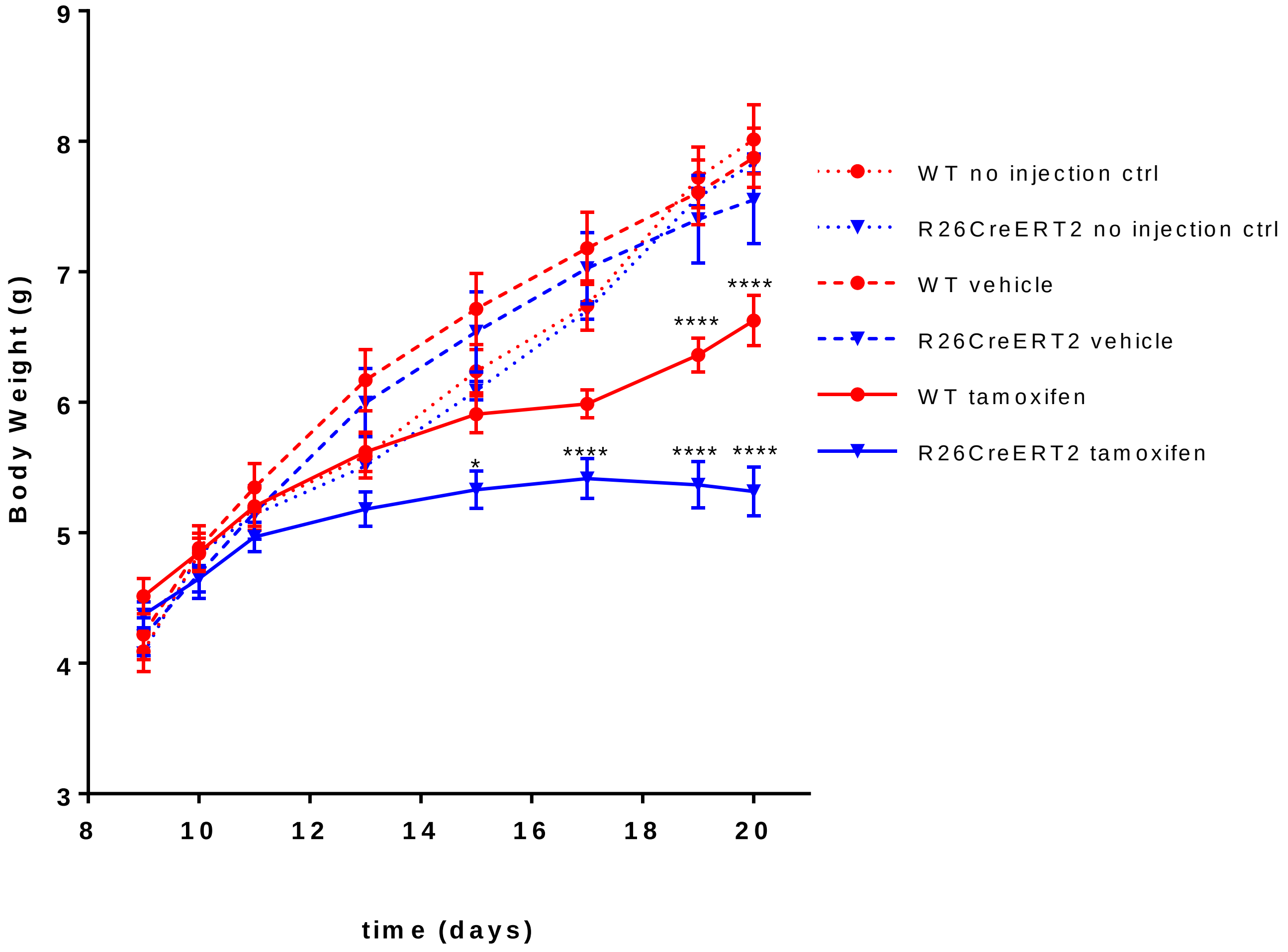

Body Weight (g)
9
8
7
6
5
4
3
8
10
12
14
16
18
20
time (days)
*
****
****
****
****
****
WT no injection ctrl
R26CreERT2 no injection ctrl
WT vehicle
R26CreERT2 vehicle
WT tamoxifen
R26CreERT2 tamoxifen

Figure S2

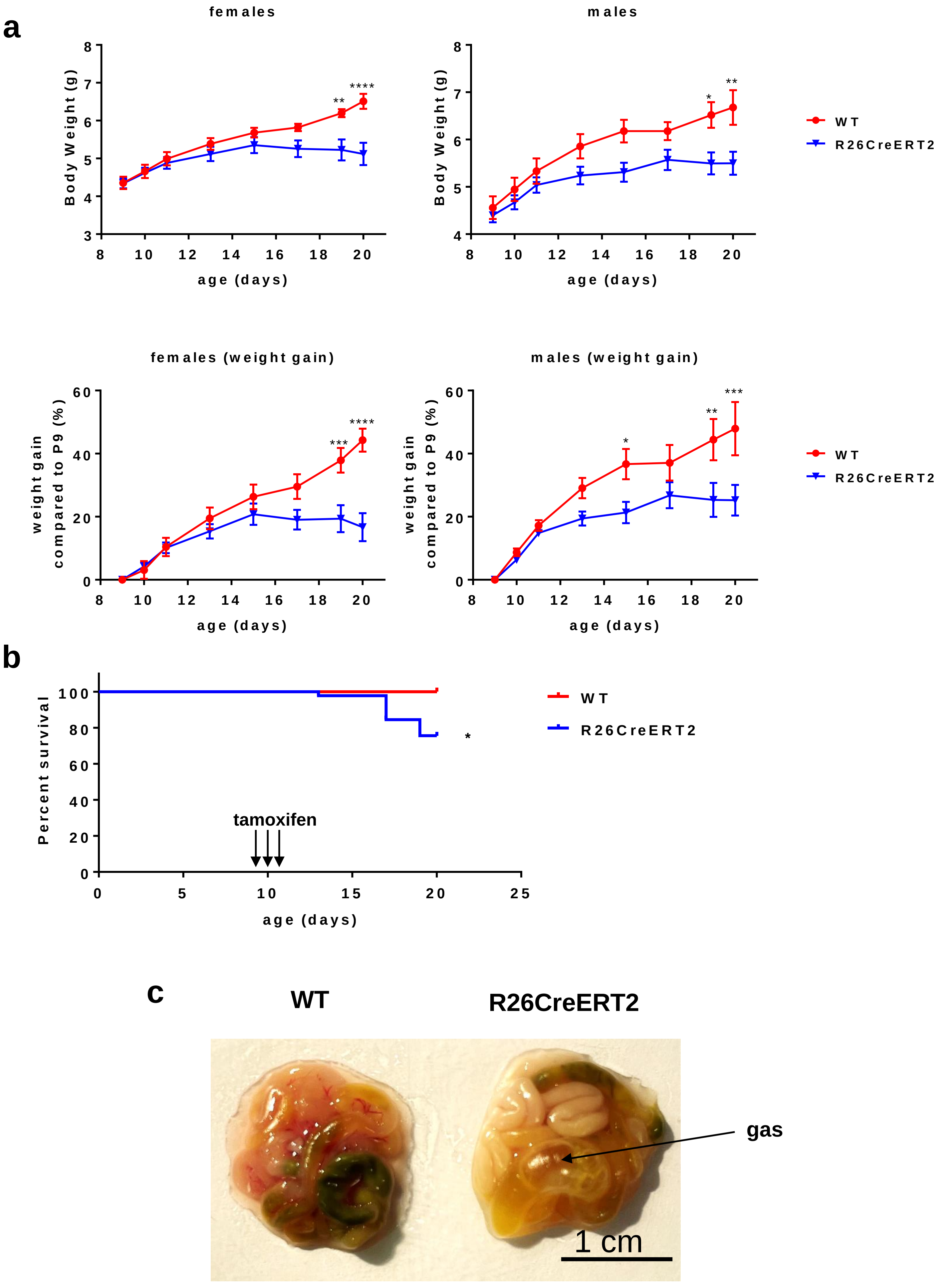
a
females
males
Body Weight (g)
age (days)
females (weight gain)
males (weight gain)
weight gain
compared to P9 (%)
WT
R26CreERT2
b
Percent survival
tamoxifen
age (days)
WT
R26CreERT2
c
WT
R26CreERT2
gas
1 cm

Figure S3

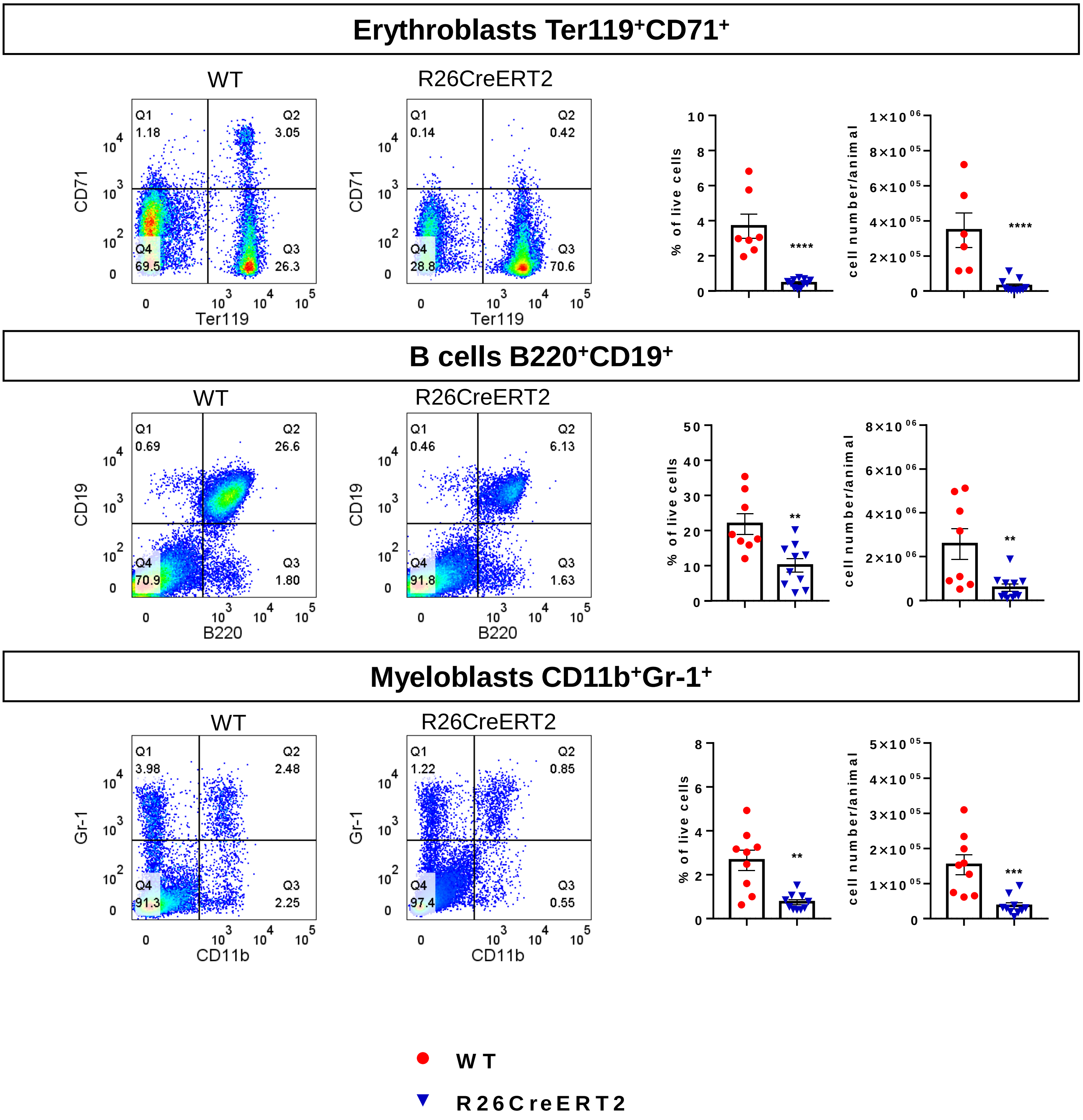

Erythroblasts Ter119⁺CD71⁺
WT
R26CreERT2
CD71
Ter119
Q1 1.18
Q2 3.05
Q4 69.5
Q3 26.3
Q1 0.14
Q2 0.42
Q4 28.8
Q3 70.6
% of live cells
cell number/animal
****
****
B cells B220⁺CD19⁺
WT
R26CreERT2
CD19
B220
Q1 0.69
Q2 26.6
Q4 70.9
Q3 1.80
Q1 0.46
Q2 6.13
Q4 91.8
Q3 1.63
% of live cells
cell number/animal
**
**
Myeloblasts CD11b⁺Gr-1⁺
WT
R26CreERT2
Gr-1
CD11b
Q1 3.98
Q2 2.48
Q4 91.3
Q3 2.25
Q1 1.22
Q2 0.85
Q4 97.4
Q3 0.55
% of live cells
cell number/animal
**
***
WT
R26CreERT2

Figure S4

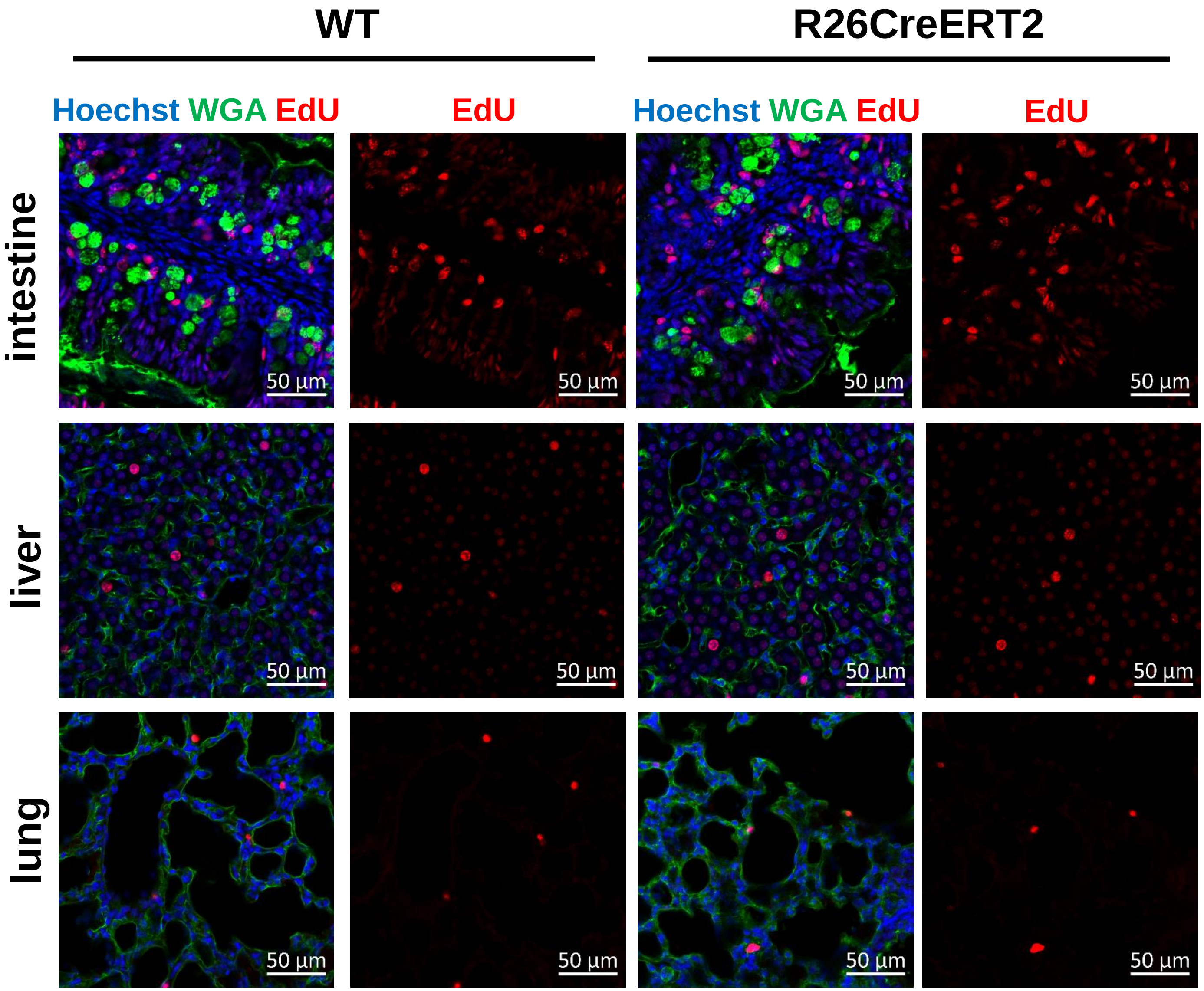
WT
R26CreERT2
Hoechst WGA EdU
EdU
Hoechst WGA EdU
EdU
intestine
liver
lung
50 µm
